# Quarnet Inference Rules for Level-1 Networks

**DOI:** 10.1007/s11538-018-0450-2

**Published:** 2018-06-04

**Authors:** Katharina T. Huber, Vincent Moulton, Charles Semple, Taoyang Wu

**Affiliations:** 1 School of Computing Sciences, University of East Anglia, Norwich, UK; 2 Biomathematics Research Centre, University of Canterbury, Christchurch, New Zealand

**Keywords:** Inference rules, Phylogenetic network, Quartet trees, Closure, Cyclic orderings, Level-1 network, Quarnet, Qnet

## Abstract

An important problem in phylogenetics is the construction of phylogenetic trees. One way to approach this problem, known as the supertree method, involves inferring a phylogenetic tree with leaves consisting of a set *X* of species from a collection of trees, each having leaf-set some subset of *X*. In the 1980s, Colonius and Schulze gave certain inference rules for deciding when a collection of 4-leaved trees, one for each 4-element subset of *X*, can be simultaneously displayed by a single supertree with leaf-set *X*. Recently, it has become of interest to extend this and related results to phylogenetic networks. These are a generalization of phylogenetic trees which can be used to represent reticulate evolution (where species can come together to form a new species). It has recently been shown that a certain type of phylogenetic network, called a (unrooted) level-1 network, can essentially be constructed from 4-leaved trees. However, the problem of providing appropriate inference rules for such networks remains unresolved. Here, we show that by considering 4-leaved networks, called quarnets, as opposed to 4-leaved trees, it is possible to provide such rules. In particular, we show that these rules can be used to characterize when a collection of quarnets, one for each 4-element subset of *X*, can all be simultaneously displayed by a level-1 network with leaf-set *X*. The rules are an intriguing mixture of tree inference rules, and an inference rule for building up a cyclic ordering of *X* from orderings on subsets of *X* of size 4. This opens up several new directions of research for inferring phylogenetic networks from smaller ones, which could yield new algorithms for solving the supernetwork problem in phylogenetics.

## Introduction

**Fig. 1:**
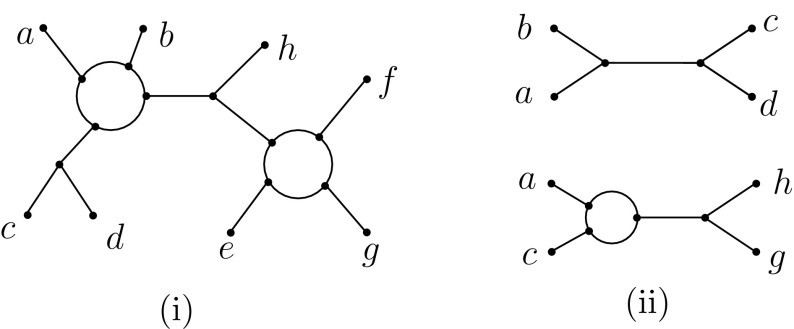
**i** A level-1 phylogenetic network with leaf-set $X=\{a,b,\ldots ,h\}$. **ii** Top: a quartet tree with leaf-set $\{a,b,c,d\}$, also denoted by *ab*|*cd*. Bottom: a quarnet with leaf-set $\{a,c,h,g\}$. Both the quartet tree and quarnet are displayed by the level-1 network in **i**

One of the main goals in phylogenetics is to develop methods for constructing evolutionary trees, the tree-of-life being a prime example of such a tree (Letunic and Bork 2016). Mathematically speaking, for a set *X* of species, a phylogenetic *X*-tree is a (graph theoretical) tree with leaf-set *X* and no degree-2 vertices; it is *binary* if every internal vertex has degree three. A popular approach to constructing such trees, called the *supertree method*, is to build them up from smaller trees (Bininda-Emonds 2014). The smallest possible trees that can be used in this approach are *quartet trees*, that is, binary phylogenetic trees having 4 leaves (see e.g. Fig. 1 for the quartet tree *ab*|*cd* with leaf-set $\{a,b,c,d\} \subseteq X$). Thus, it is natural to ask the following question: How should we decide whether or not it possible to simultaneously display all of the quartet trees in a given collection $\mathcal {Q}$ of quartet trees by some phylogenetic tree?

In case the collection $\mathcal {Q}$ consists of a quartet tree for every possible subset of *X* of size 4 (which we denote by $X \atopwithdelims ()4$), this problem has an elegant solution that was originally presented by Colonius and Schulze (1981) (see also Bandelt and Dress 1986 for related results). We present full details in Theorem 1, but essentially their result states that, given a collection of quartet trees $\mathcal {Q}$, one for each element in $X \atopwithdelims ()4$, there exists (a necessarily unique) binary phylogenetic *X*-tree displaying every quartet tree in the collection if and only if when the quartet trees *ab*|*cx* and *ab*|*xd* are contained in $\mathcal {Q}$ then so is the quartet tree *ab*|*cd*. Rules such as *ab*|*cx* plus *ab*|*xd* implies *ab*|*cd* are known as *inference rules*, and they have been extensively studied in the phylogenetics literature (see e.g. Semple and Steel 2003, Chapter 6.7 and the references therein).

Although phylogenetic trees are extremely useful for representing evolutionary histories, in certain circumstances they can be inadequate. For example, when two viruses recombine to form a new virus (e.g. swine flu), this is not best represented by a tree as it involves species combining together to form a new one rather than splitting apart. In such cases, phylogenetic networks provide a more accurate alternative to trees and there has been much recent work on such structures (see e.g. Steel 2016, Chapter 10 for a recent review).

In this paper, we will consider properties of a particular type of phylogenetic network called a *level-1 network* (Gambette et al. 2012).^1^ For a set *X* of species, this is a connected graph with leaf-set *X* and such that every maximal subgraph with no cut-edge is either a vertex or a cycle (see Sect. 2 for more details). Our main results will apply to binary level-1 networks, where we also assume that every vertex has degree 1 or 3. We present an example of such a network in Fig. 1. Note that a phylogenetic *X*-tree is a special example of a level-1 network with leaf-set *X*. As with phylogenetic *X*-trees, it is possible to construct level-1 networks from quartets (Gambette et al. 2012). However, it has been pointed out that there are problems with understanding such networks in terms of inference rules (see e.g. Keijsper and Pendavingh 2014, p. 2540).

Here, we circumvent these problems by considering a certain type of subnetwork of level-1 network called a *quarnet* instead of using quartet trees. A quarnet is a 4-leaved, binary, level-1 network (see e.g. Fig. 1); they are displayed by binary level-1 networks in a similar way to quartets (see Sect. 3 for details). As we shall see, quarnets naturally lead to inference rules for level-1 networks which can be thought of as a combination of quartet inference and inference rules for building circular orderings of a set. Moreover, in our main result we show that, just as with phylogenetic trees, the quarnet inference rules that we introduce can be used to characterize when a collection of quarnets, one for each element in ${X \atopwithdelims ()4}$, is equal to the set of quarnets displayed by a binary level-1 network with leaf-set *X*.

We now summarize the contents of the rest of the paper. In the next section, we present some preliminaries concerning phylogenetic trees and level-1 networks, as well as their relationship with quartets. Then, in Sect. 3, we prove an analogous theorem to the quartet results of Colonius and Schulze for level-1 networks (Theorem 2). In Sect. 4, we use Theorem 2 to provide a characterization for when a set of quartets, one for each element of $X \atopwithdelims ()4$, can be displayed by a binary level-1 network (Theorem 3). In Sect. 5, we then define the closure of a set of quarnets. This can be thought of as the collection of quarnets that is obtained by applying inference rules to a given collection of quarnets until no further quarnets are generated. We show that this has similar properties to the so-called *semi-dyadic closure* of a set of quartets (see Theorem 4). We conclude with a brief discussion of some possible further directions.

## Preliminaries

In this section, we review some definitions as well as results concerning the connection between phylogenetic trees and quartets. From now on, we assume that *X* is a finite set with $|X|\ge 2$.

### Definitions

An *unrooted phylogenetic network N (on X)* (or *network N (on X)* for short) is a connected graph (*V*, *E*) with $X \subseteq V$, every vertex has either degree 1 or degree at least 3, and the set of degree-1 vertices is *X*. The elements in *X* are the *leaves* of *N*. We also denote the leaf-set of *N* by *L*(*N*). The network is called *binary* if every vertex in *N* has degree 1 or 3. An *interior vertex* of *N* is a vertex that is not a leaf. A *cherry* in *N* is a pair of leaves that are adjacent with the same vertex. Two phylogenetic networks *N* and $N'$ on *X* are *isomorphic* if there exists a graph theoretical isomorphism between *N* and $N'$ whose restriction to *X* is the identity map.

Note that a *phylogenetic (X-) tree* is a network which is also a tree. For any three vertices $u_1,u_2,u_3$ in such a tree *T*, their *median*, denoted by $\mathop {med}(u_1,u_2,u_3)=\mathop {med}_T(u_1,u_2,u_3)$, is the unique vertex in *T* that is contained in every path between any two vertices in $\{u_1, u_2, u_3\}$.

A *cut-vertex* of a network is a vertex whose removal disconnects the network, and a *cut-edge* of a network is an edge whose removal disconnects the network. A cut-edge is *trivial* if one of the connected components induced by removing the cut-edge is a vertex (which must necessarily be a leaf). A network is *simple* if all of the cut-edges are trivial (so for instance, note that phylogenetic trees with more than three leaves are *not* simple networks). A network *N* is *level-1* if every maximal subgraph in *N* that has no cut-edge is either a vertex or a cycle. Note that we shall say that a network *N* on *X*, where $|X|\ge 3$, is of *cycle type* if it contains a unique cycle of length |*X*|, and the number of vertices in *N* is 2|*X*| (so in particular, a network is of cycle type if it is simple, binary, level-1 and is not a phylogenetic tree).

**Fig. 2:**
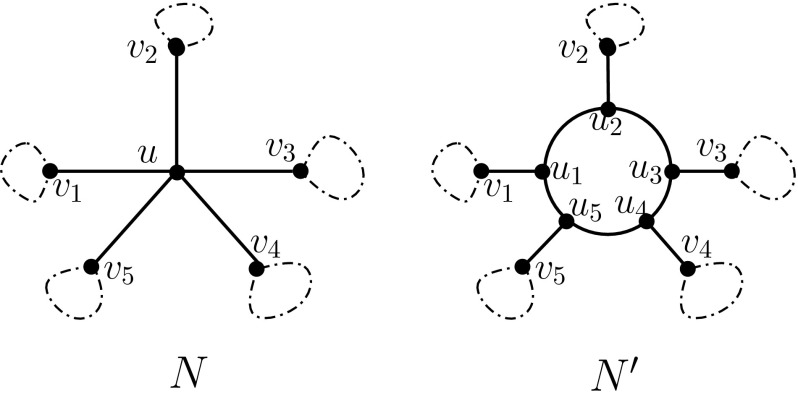
Example of blow-up operations: $N'$ is obtained from *N* by a blow-up operation on *u*

In what follows it will be useful to consider a certain type of operation on a level-1 network, which we define as follows. For a level-1 network *N* on *X*, let *u* be an interior vertex of *N* that is not contained in any cycle in *N*. Furthermore, let $(v_1, v_2, \ldots ,v_k)$, where $k\ge 3$, be a circular ordering of the set of vertices in *N* that are adjacent to *u*. Then, we obtain a new network $N'$ on *X* from *N* by removing vertex *u* and all edges incident with it and inserting new vertices $u_i$ and new edges $\{u_i,v_i\}$ and $\{u_i, u_{i+1}\}$ for all $1\le i \le k$ (see Fig. 2). Here, we use the convention that $k+1$ is identified with 1. We say that $N'$ is obtained from *N* by a *blow-up* operation on *u* (using the given circular ordering of its neighbours). Note that $N'$ is a level-1 network with one more cycle than *N*. Note that blow-up operations on the same vertex but with different circular orderings of its neighbours may lead to non-isomorphic networks. We illustrate a blow-up operation in Fig. 2.

### Quartets, Trees and Networks

We now briefly recall some notation and results concerning quartet systems (for more details see Dress et al. 2012, Chapter 3).

Although quartets are often considered as being 4-leaved trees, here it is more convenient to consider a *quartet* *Q* to be a partition of a subset *Y* of *X* of size 4 into two subsets of size 2. The set *Y* is called the *support* of *Q*. If $Q = \{\{a,b\},\{c,d\}\}$ for $a,b,c,d \in X$ distinct, we denote *Q* by *ab*|*cd*. The set of all quartets on *X* is denoted by ${\mathcal {Q}}(X)$, and any non-empty subset ${\mathcal {Q}}\subseteq {\mathcal {Q}}(X)$ is called a *quartet system* (on *X*). Given a quartet system ${\mathcal {Q}}$ on *X* and a subset $Y\in {X \atopwithdelims ()4 }$, let $m(Y)=m_{{\mathcal {Q}}}(Y)$ be the number of quartets in ${\mathcal {Q}}$ whose support is *Y*. For simplicity, we write $m(\{a,b,c,d\})$ as *m*(*a*, *b*, *c*, *d*). If $m(Y)\ge 1$ for every subset $Y\in {X \atopwithdelims ()4 }$, then ${\mathcal {Q}}$ is said to be *dense*.

Following the terminology in Dress et al. (2012), a quartet system ${\mathcal {Q}}$ is:

- *thin* if no pair of quartets in ${\mathcal {Q}}$ have the same support;
- *saturated* if for all $\{a,b,c,d,x\}\in {X \atopwithdelims ()5}$ with $ab|cd \in {\mathcal {Q}}$, the system ${\mathcal {Q}}$ contains at least one quartet in $\{ax|cd, ab|cx\}$;
- *transitive* if for all $\{a,b,c,d,x\}\in {X \atopwithdelims ()5}$, if $\{ab|cx, ab|xd\}\subseteq {\mathcal {Q}}$ holds, then *ab*|*cd* is also contained in ${\mathcal {Q}}$.

These concepts are related as follows:

#### Lemma 1

Suppose that ${\mathcal {Q}}$ is a quartet system on *X*. If ${\mathcal {Q}}$ is saturated and thin, then ${\mathcal {Q}}$ is transitive.

#### Proof

We use a similar argument to that used by Bandelt and Dress (1986, Lemma 1). Suppose $\{a,b,c,d,x\}\in {X \atopwithdelims ()5}$ with $\{ab|cx, ab|xd\}\subseteq {\mathcal {Q}}$. We need to show $ab|cd\in {\mathcal {Q}}$.

Since ${\mathcal {Q}}$ is saturated and *ab*|*cx* is contained in ${\mathcal {Q}}$, we have $\{ab|cd, ad|cx\}\cap {\mathcal {Q}}\not = \emptyset$. Using a similar argument, *ab*|*dx* in ${\mathcal {Q}}$ implies that $\{ab|cd, ac|dx\}\cap {\mathcal {Q}}\not = \emptyset$. Therefore, we must have $ab|cd\in {\mathcal {Q}}$ as otherwise $\{ad|cx,ac|dx\}\subset {\mathcal {Q}}$, a contradiction to the assumption that ${\mathcal {Q}}$ is thin. $\square$

A quartet *ab*|*cd* on *X* is *displayed* by a phylogenetic *X*-tree *T* if the path between *a* and *b* in *T* is vertex disjoint from the path between *c* and *d* in *T*. The quartet system displayed by *T* is denoted by ${\mathcal {Q}}(T)$.

In view of Dress et al. (2012, Theorem 3.7) and the last lemma, we have the following slightly stronger characterisation of quartet systems displayed by a phylogenetic tree, which was stated in Bandelt and Dress (1986, Proposition 2) using slightly different terminology.

#### Theorem 1

A quartet system ${\mathcal {Q}}\subseteq {\mathcal {Q}}(X)$ is of the form ${\mathcal {Q}}={\mathcal {Q}}(T)$ for a (necessarily unique) phylogenetic *X*-tree *T* if and only if ${\mathcal {Q}}$ is thin and saturated.

We now turn our attention to the relationship between quartets and level-1 networks.

A split *A*|*B* of *X* is a bipartition of *X* into two non-empty parts *A* and *B* (note that since *A*|*B* is a bipartition, order does not matter, that is, $A|B=B|A$). Such a split is induced by a network *N* if there exists a cut-edge in *N* whose removal results in two connected components, one with leaf-set *A* and the other with leaf-set *B*. A quartet *ab*|*cd* is *exhibited* by a network *N* if there exists a split *A*|*B* induced by *N* such that $\{a,b\}\subseteq A$ and $\{c,d\}\subseteq B$.

Note that if a quartet $ab|cd \in {\mathcal {Q}}(X)$ is exhibited by *N*, then it is *displayed* by *N*, that is, *N* contains two disjoint paths, one from *a* to *b*, and the other from *c* to *d*. However, the converse is not true. For example, quartet *ab*|*cd* is displayed by the network in Fig. 4(iv), but *ab*|*cd* is not exhibited by this network. Given a network *N*, we let $\Sigma (N)$ denote the set of quartets exhibited by *N*, and let ${\mathcal {Q}}(N)$ be the set of quartets displayed by *N*. In the light of the last remark, clearly we have $\Sigma (N)\subseteq {\mathcal {Q}}(N)$.

## Quarnets

In this section, we shall show that an analogue of Theorem 1 holds for quarnets and level-1 networks. We begin by formally defining the concept of a quarnet and how quarnets can be obtained from level-1 networks.

Given a binary, level-1 phylogenetic network *N* on *X* and a subset $A\subseteq X$, we let $N|_A$ denote the *network induced on A by N*, which is obtained from *N* by deleting all edges that are not contained in some path between a pair of elements in *A*, removing all isolated vertices, and then repeatedly applying the following two operations until neither of them is applicable (i) suppressing degree-2 vertices, and (ii) suppressing parallel edges. Note that $N|_A$ is a binary, level-1 phylogenetic network on *A*.

**Fig. 3:**
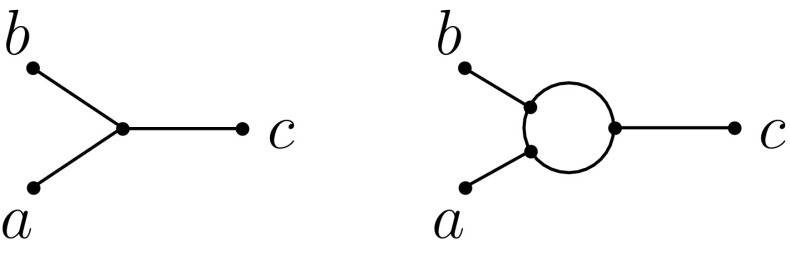
The two types of three-leaved networks: tree type (left) and cycle type (right)

**Fig. 4:**
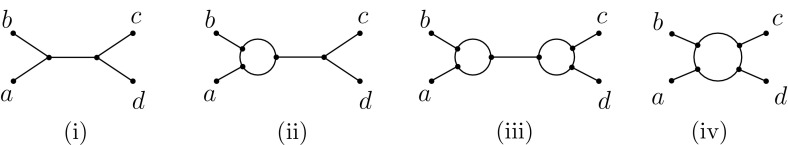
Four types of qnets on $X=\{a,b,c,d\}$: **i** a Type I qnet ${a}\ominus {b}|{c} \ominus {d}$; **ii** a Type II qnet ${a}\oplus {b}|{c}\ominus {d}$; **iii** a type III qnet ${a}\oplus {b}|{c}\oplus {d}$; **iv** a type IV qnet ${a}\oplus {b}\oplus {c}\oplus {d}$. Type IV is of cycle type

We now consider the different possible phylogenetic networks on three and four leaves. First note that there are two possible types of phylogenetic networks with three leaves (see Fig. 3). We call these *cycle type* and *tree type* depending on whether they contain a cycle or not, respectively. Similarly, a *quarnet* or *qnet, for short*,^2^ is a binary, level-1 phylogenetic network with four leaves. The leaf-set *L*(*F*) of a qnet *F* is called its *support*. As illustrated in Fig. 4, there are four types of qnets: Type I qnets contain no cycles; Type II qnets contain one cycle and one non-trivial cut-edge; Type III qnets contain two cycles; and Type IV qnets contain no non-trivial cut-edge. A *qnet system* ${\mathcal {F}}$ on *X* is a collection of qnets all of whose supports are contained in *X*. We shall say that a qnet *F* with support $A \subseteq X$ is *displayed* by a network *N* on *X* if *F* is isomorphic to $N|_A$. Moreover, we let ${\mathcal {F}}(N)$ be the qnet system displayed by *N*, that is,

$$\begin{aligned} {\mathcal {F}}(N)=\{N|_A\,~~\text{ for } \text{ all } A\subseteq X \text{ with } |A|=4\}. \end{aligned}$$

We now turn to characterizing when a qnet system is displayed by a level-1 network. To do this, we introduce some additional concepts concerning qnet systems.

First, a qnet system ${\mathcal {F}}$ on *X* is *consistent* (on subsets of *X* of size three) if for all subsets $A \in {X \atopwithdelims ()3}$, $F|_A$ is isomorphic to $F'|_A$, for each pair of qnets in ${\mathcal {F}}$ with $A\subseteq L(F)\cap L(F')$. In addition, a qnet system ${\mathcal {F}}$ on *X* is *minimally dense* if for all $Y \in {X \atopwithdelims ()4}$, there exists precisely one qnet in ${\mathcal {F}}$ with support *Y*.

Second, we say that a qnet system ${\mathcal {F}}$ on *X* is *cyclically transitive or cyclative* if for all subsets $\{a,b,c,d,x\} \in {X \atopwithdelims ()5}$ with $\{{a}\oplus {b}\oplus {c}\oplus {d}, {x}\oplus {a}\oplus {c}\oplus {d}\} \subseteq {\mathcal {F}}$, the system ${\mathcal {F}}$ also contains ${a}\oplus {b}\oplus {d}\oplus {x}$. Note that this is closely related to the cyclic-ordering inference rule given in Bandelt and Dress (1992, Proposition 1).

Finally, we say that a qnet system ${\mathcal {F}}$ on *X* is *saturated*, if for all subsets $\{a,b,c,d,x\} \in {X \atopwithdelims ()5}$, the following hold:

1. If ${\mathcal {F}}$ contains ${a}\ominus {b}|{c} \ominus {d}$, then ${a}\ominus {b}|{c} \ominus {x}$, or ${a}\ominus {b}|{c}\oplus {x}$, or ${a}\ominus {x}|{c} \ominus {d}$, or ${a}\oplus {x}|{c}\ominus {d}$ is contained in ${\mathcal {F}}$.
2. If ${\mathcal {F}}$ contains ${a}\oplus {b}|{c}\ominus {d}$, then ${a}\oplus {b}|{c}\ominus {x}$, or ${a}\oplus {b}|{c}\oplus {x}$, or ${a}\ominus {x}|{c} \ominus {d}$, or ${a}\oplus {x}|{c}\ominus {d}$ is contained in ${\mathcal {F}}$.
3. If ${\mathcal {F}}$ contains ${a}\oplus {b}|{c}\oplus {d}$, then ${a}\oplus {b}|{c}\ominus {x}$, or ${a}\oplus {b}|{c}\oplus {x}$, or ${a}\ominus {x}|{c}\oplus {d}$, or ${a}\oplus {x}|{c}\oplus {d}$ is contained in ${\mathcal {F}}$.

We next show how these concepts are related. To prove the following result, given a qnet system ${\mathcal {F}}$, we shall consider the quartet system consisting of those quartets that are exhibited by some qnet in ${\mathcal {F}}$, which we shall denote by $\Sigma ({\mathcal {F}})$.

### Lemma 2

Suppose that ${\mathcal {F}}$ is a qnet system on *X*.

(i) If ${\mathcal {F}}$ is minimally dense, then $\Sigma ({\mathcal {F}})$ is thin.
(ii) If ${\mathcal {F}}$ is saturated, then $\Sigma ({\mathcal {F}})$ is saturated.

### Proof

For the proof of (i), as ${\mathcal {F}}$ is minimally dense, for each subset *Y* of *X* with size four, there exists precisely one qnet *F* in ${\mathcal {F}}$ whose support is *Y*. Hence, there exists at most one quartet in $\Sigma ({\mathcal {F}})$ with support *Y*.

To prove (ii), consider a quartet $Q=ab|cd$ in ${\mathcal {Q}}({\mathcal {F}})$ and an arbitrary element *x* in *X* that is distinct from *a*, *b*, *c*, *d*. Let *F* be a qnet in ${\mathcal {F}}$ such that *Q* is the quartet exhibited by *F*. Then, *F* is Type I, II or III. Assume first that *F* is Type I, then $F={a}\ominus {b}|{c} \ominus {d}$. Since ${\mathcal {F}}$ is saturated, by (S1),

$$\begin{aligned} \{{a}\ominus {b}|{c} \ominus {x}, {a}\ominus {b}|{c}\oplus {x}, {a}\ominus {x}|{c} \ominus {d}, {a}\oplus {x}|{c}\ominus {d}\} \cap {\mathcal {Q}}\not = \emptyset , \end{aligned}$$

and so one of the quartets *ab*|*cx* and *ax*|*cd* is contained in $\Sigma ({\mathcal {F}})$, as required. If *F* is of Type II or III, then similar arguments using (S2) and (S3), respectively, show that *ab*|*cx* or *ax*|*cd* is contained in $\Sigma (\mathcal {F})$. $\square$

We now characterize when a minimally dense set of qnets is displayed by a level-1 network.

### Theorem 2

Let ${\mathcal {F}}$ be a minimally dense qnet system on *X* with $|X|\ge 4$. Then, ${\mathcal {F}}={\mathcal {F}}(N)$ for some (necessarily unique) binary, level-1 network *N* on *X* if and only if ${\mathcal {F}}$ is consistent, cyclative and saturated.

### Proof

Clearly, if ${\mathcal {F}}={\mathcal {F}}(N)$ holds for a binary, level-1 network *N*, then ${\mathcal {F}}(N)$ is consistent, cyclative and saturated.

We now show that the converse holds. Suppose that ${\mathcal {F}}$ is a minimally dense qnet system on *X* that is consistent, cyclative and saturated. Consider the quartet system $\Sigma =\Sigma ({\mathcal {F}})$. By Lemma 2, $\Sigma$ is thin and saturated. Therefore, by Theorem 1, there exists a unique phylogenetic tree *T* with ${\mathcal {Q}}(T)=\Sigma$.

For each interior vertex *v* in *T*, let ${\mathcal {A}}_v$ denote the partition of *X* induced by deleting *v* from *T* so that, in particular, the number of parts in ${\mathcal {A}}_v$ is equal to the degree of *v*. Note that, for all $A \in {\mathcal {A}}_v$, if $a \in A$ and $b\in X-A$, the path in *T* between *a* and *b* must contain *v*, and if $a,b \in A$, the path between *a* and *b* does not contain *v*.

We next partition the set of interior vertices of *T*. Let $V_1(T)$ be the set of degree-3 vertices *v* in *T* with the property that there exist three elements, one from each distinct part of ${\mathcal {A}}_v$, so that there exists a qnet *F* in ${\mathcal {F}}$ whose restriction to these three elements is of cycle type. Let $V_0(T)$ be the set of degree-3 vertices in *T* not contained in $V_1(T)$. Lastly, let $V_2(T)$ be the set of interior vertices in *T* with degree at least 4.$\square$

### Claim 1

A degree-3 vertex *v* in *T* is contained in $V_1(T)$ if and only if, for each subset *Y* of *X* of size three that contains precisely one element from each part of ${\mathcal {A}}_v$, the restriction $F|_{Y}$ is of cycle type for every qnet *F* in ${\mathcal {F}}$ with $Y\subset L(F)$.

### Proof

Since ${\mathcal {F}}$ is minimally dense, the “if ” direction follows directly from the definition of $V_1(T)$.

Conversely, let $Y^*=\{a^*_1,a^*_2,a^*_3\}$ be such that $a^*_i$, $1\le i \le 3$, are all contained in distinct parts of ${\mathcal A}_v$ and there exists a qnet $F^*$ in ${\mathcal {F}}$ such that $F^*|_{Y^*}$ is of cycle type. Now let $Y=\{a_1,a_2,a_3\}$ with $a_i$ all contained in distinct parts of ${\mathcal {A}}_v$ and let *F* be an arbitrary qnet in ${\mathcal {F}}$ with $Y\subset L(F)$. We shall show that $F|_Y$ is of cycle type by considering the size of the intersection $Y\cap Y^*$.

First assume that $|Y\cap Y^*|=3$, that is, $Y=Y^*$. Then, as ${\mathcal {F}}$ is consistent, $F|_Y$ is of cycle type since it is isomorphic to $F^*|_{Y^*}$.

Second assume that $|Y\cap Y^*|=2$. By swapping the indices, we may further assume that $a_1=a^*_1$, $a_2=a^*_2$, and $a_3\not =a^*_3$. In other words, we have $Y=\{a^*_1,a^*_2,a_3\}$. Consider $Y'=\{a^*_1,a^*_2,a_3,a^*_3\}$ and let $F'$ be the qnet in ${\mathcal {F}}$ with $L(F')=Y'$. Since $a_3,a^*_3$ are both contained in $A_v$, the quartet $Q'=a^*_1a^*_2|a_3a^*_3$ is contained in ${\mathcal {Q}}(T)$. As $F'|_{Y^*}$ is of cycle type, this implies that $F'$ is either ${a^*_1}\oplus {a^*_2}|{a_3}\ominus {a^*_3}$ or ${a^*_1}\oplus {a^*_2}\oplus {a_3}\oplus {a^*_3}$. In both cases, $F'|_{Y}$ is of cycle type, and hence $F|_Y$ is also of cycle type in view of the consistency of ${\mathcal {F}}$.

Next assume that $|Y\cap Y^*|=0$. By swapping the indices, we may further assume that, for $1\le i \le 3$, elements $a_i$ and $a^*_i$ are contained in the same part of ${\mathcal {A}}_v$ but $a_i\not = a^*_i$. Consider the sets $Y_1=\{a^*_1,a^*_2,a_3\}$ and $Y_2=\{a^*_1,a_2,a_3\}$, and put $Y_0=Y^*$ and $Y_3=Y$. Then, we have $|Y_i\cap Y_{i+1}|=2$ for $0\le i \le 2$. Repeatedly applying the argument used when the size of the intersection is two, it follows that $F|_Y$ is of cycle type, as required.

Lastly, the case $|Y\cap Y^*|=1$ can be established using a similar argument to that when the size of the intersection is zero. This completes the proof of the claim. $\square$

Although we will not use this fact later, note that it follows from Claim 1 that a vertex *v* in *T* is contained in $V_0(T)$ if and only if, for each subset *Y* of *X* of size three whose elements are contained in distinct elements of $\mathcal {A}_v$, the restriction $F|_Y$ is a tree type for every qnet *F* in $\mathcal {F}$ with $Y\subset L(F)$.

### Claim 2

Suppose $v \in V_2(T)$. Let $x,y,p,q \in X$ be contained in distinct parts $A_x,A_y,A_p,A_q$ of $\mathcal {A}_v$, respectively. Then, the qnet *F* in ${\mathcal {F}}$ with support $A = \{x,y,p,q\}$ is of Type IV. Moreover, if *F* is ${x}\oplus {y}\oplus {p}\oplus {q}$, then, for all $x'\in A_x$, $y' \in A_y$, $p'\in A_p$ and $q'\in A_q$, the qnet $F'$ with support $A' = \{x',y',p',q'\}$ is ${x'}\oplus {y'}\oplus {p'}\oplus {q'}$.

### Proof

Suppose *F* is not of Type IV. Then, $\Sigma (F)$ contains precisely one quartet, denoted by *Q*, and $L(Q)=A$. This implies that $Q \in \Sigma ({\mathcal {F}})={\mathcal {Q}}(T)$. However, *Q* is not contained in ${\mathcal {Q}}(T)$ because the path between any pair of distinct elements in *A* contains *v*; a contradiction. Thus, *F* is of Type IV.

Now, suppose $|A\cap A'|=3$. Then, we may further assume without loss of generality that $x=x'$, $y=y'$, $p=p'$, and $q\not = q'$. Hence, $A'=\{x,y,p,q'\}$. Note that the argument in the last paragraph implies that $F'$ is of Type IV. If $F'$ is not isomorphic to ${x}\oplus {y}\oplus {p}\oplus {q'}$, then $F'$ is isomorphic to either ${x}\oplus {y}\oplus {q'}\oplus {p}$ or ${x}\oplus {p}\oplus {y}\oplus {q'}$. In the first subcase, since ${\mathcal {F}}$ is cyclative and $\{{x}\oplus {y}\oplus {p}\oplus {q}, {x}\oplus {y}\oplus {q'}\oplus {p}\} \subset {\mathcal {F}}$, the qnet ${p}\oplus {q}\oplus {y}\oplus {q'}$ is contained in ${\mathcal {F}}$. This implies that the quartet $Q'= py|q q'$ is not contained in ${\mathcal {Q}}(T)$, a contradiction since $q,q'$ are contained in $A_q$ while *p*, *y* are contained in $X-A_q$. The second subcase follows in a similar way.

Lastly, if $|A\cap A'|\le 2$, then note that there exists a list of 4-element subsets $A=A_0,\ldots ,A_t=A'$ for some $t\ge 1$ such that, for $0\le i <t$, we have $|A_i \cap A_{i+1}|=3$ and the two elements in $(A_i-A_{i+1})\cup (A_{i+1}-A_i)$ are contained in the same part of $\mathcal {A}_v$. Claim 2 follows by repeatedly applying the argument in the last paragraph to the list. $\square$

Using the last claim, we next establish the following.

### Claim 3

For each vertex $v \in V_2(T)$, there exists a unique circular ordering of the parts $A^1,\ldots ,A^m$ of $\mathcal {A}_v$ such that, for each tuple $A=(a_i,a_j,a_k,a_l) \in A^i \times A^j \times A^k \times A^l$ with $1\le i<j<k<l \le m$, the qnet in ${\mathcal {F}}$ with support $\{{a_i,a_j,a_k,a_l}\}$ is isomorphic to ${a_i}\oplus {a_j}\oplus {a_k}\oplus {a_l}$.

### Proof

In the light of Claim 2, we can define a quaternary relation || on the parts of $\mathcal {A}_v$ by setting *AB*||*CD*, for all distinct parts $A,B,C,D \in \mathcal {A}_v$, if and only if, for all $x \in A$, $y \in B$, $p \in C$ and $q \in D$, the qnet with support $\{x,y,p,q\}$ is ${x}\oplus {p}\oplus {y}\oplus {q}$. Put differently, the distance between *x* and *p* in the qnet with support $\{x,y,p,q\}$ is two, and so is the distance between *y* and *q*.

Now, for all distinct $A,B,C,D,E \in \mathcal {A}_v$, we show that

1. *AB*||*CD* implies *BA*||*CD* and *CD*||*AB*;
2. either *AB*||*CD*, or *AC*||*BD*, or *AD*||*BC* (exclusively);
3. *AC*||*BD* and *AD*||*CE* implies *AC*||*BE*.

Indeed, let $x \in A$, $y \in B$, $p \in C$, $q \in D$, $r \in E$. Then, (BD-1) holds since ${x}\oplus {p}\oplus {y}\oplus {q}$ is isomorphic to ${y}\oplus {p}\oplus {x}\oplus {q}$ and to ${p}\oplus {x}\oplus {q}\oplus {y}$. Next, (BD-2) follows immediately since $\mathcal {F}$ is minimally dense. To see (BD-3) holds, note that since *AD*||*CE* and *AC*||*BD* imply that ${x}\oplus {r}\oplus {q}\oplus {p}$ and ${x}\oplus {q}\oplus {p}\oplus {y}$ are contained in ${\mathcal {F}}$, using the fact that ${\mathcal {F}}$ is cyclative implies that ${x}\oplus {r}\oplus {p}\oplus {y}$ is in ${\mathcal {F}}$, and hence *AC*||*EB* holds. Using (BD-1), it follows that *AC*||*BE*, as required.

Since the quaternary relation || on $\mathcal {A}_v$ satisfies the conditions (BD-1)–(BD-3) as specified in Proposition 1 on page 73 of Bandelt and Dress (1992), it follows that || determines a unique circular ordering of the parts in $\mathcal {A}_v$ as specified in Claim 3. $\square$

Now let $V'=V_1(T)\cup V_2(T)$, and for each vertex $u\in V'$, fix a circular ordering of its neighbourhood $N_u(T)$ induced by the ordering of $\mathcal {A}_u$ in Claim 3 if $u\in V_2(T)$, or the necessarily unique circular ordering (clockwise and anticlockwise are treated as the same) of $N_u(T)$ if $u\in V_1(T)$ (and hence $|N_u(T)| =3$). Let *N* be the level-1 network obtained from *T* by blowing up each vertex *u* in $V'$ using the given circular ordering of $N_u(T)$. We next show that ${\mathcal {F}}\subseteq {\mathcal {F}}(N)$. To this end, fix four arbitrary elements *a*, *b*, *c*, *d* in *X* and let *F* be the qnet in ${\mathcal {F}}$ with support $\{a,b,c,d\}$. We need to show that $F\in {\mathcal {F}}(N)$. There are four cases depending upon whether *F* is Type I, II, III, or IV.

First suppose *F* is of Type I. Without loss of generality, we may assume that $F={a}\ominus {b}|{c} \ominus {d}$. Let $u=\mathop {med}_T(a,b,c)$. If $u\in V_1(T)\cup V_2(T)$, then *a*, *b*, *c* are contained in three distinct parts in the partition ${\mathcal {A}}_u$ of *X* on *u*. By Claims 1 and 2, it follows that $F|_A$ with $A=\{a,b,c\}$ is of cycle type, a contradiction. Thus, $u\in V_0(T)$ and so there exists a cut-vertex in *N* whose removal induces three connected components, containing *a*, *b* and *c*, respectively. Similarly, the median $v=\mathop {med}_T(a,c,d)$ is contained in $V_0(T)$. Hence, there exists a cut-vertex in *N* whose removal induces three connected components, containing *a*, *c* and *d*, respectively. Let $F'$ be the qnet in ${\mathcal {F}}(N)$ whose support is $\{a,b,c,d\}$. Thus, by inspecting all possible qnets on $\{a,b,c,d\}$, it follows that $F'$ is isomorphic to ${a}\ominus {b}|{c} \ominus {d}$, and hence $F\in {\mathcal {F}}(N)$.

Second, suppose that *F* is of Type II. Without loss of generality, we may assume that $F={a}\oplus {b}|{c}\ominus {d}$. Let $F'$ be the qnet in ${\mathcal {F}}(N)$ whose support is $\{a,b,c,d\}$. Let *u* be the median of *a*, *c*, *d* in *T*. Then, by an argument similar to the one used in the last paragraph, it follows that there exists a cut-vertex in *N* (and hence also a cut-vertex in $F'$) whose removal results in three connected components, containing *a*, *c* and *d*, respectively. On the other hand, let *v* be the median of $A=\{a,b,c\}$ in *T*. Then, *a*, *b*, *c* are contained in three distinct parts of ${\mathcal {A}}_v$. Since $F|_A$ is of cycle type, by Claim 2 it follows that $v\in V_1(T)\cup V_2(T)$, which implies that $F'|_A$ is also of cycle type. Thus, by inspecting all possible qnets on $\{a,b,c,d\}$, it follows that $F'$ is isomorphic to ${a}\oplus {b}|{c}\ominus {d}$, and hence $F\in {\mathcal {F}}(N)$.

Next, suppose that *F* is of Type III. Without loss of generality, we may assume that $F={a}\oplus {b}|{c}\oplus {d}$. Let $F'$ be the qnet in ${\mathcal {F}}(N)$ whose support is $\{a,b,c,d\}$. Let *u* be the median of $A=\{a,b,c\}$ in *T* and *v* be the median of $B=\{a,c,d\}$ in *T*. Since the quartet *ab*|*cd* is contained in ${\mathcal {Q}}(T)$, we know that *u* and *v* are distinct. Hence, there exists a cut-edge whose deletion puts *a* and *b* in one component and *c* and *d* in the other connected component. By an argument similar to that used for analysing when *F* is of Type II, it follows that $F'|_A$ and $F'|_B$ are both of cycle type. Hence, by inspecting all possible qnets on $\{a,b,c,d\}$, the qnet $F'$ is isomorphic to ${a}\oplus {b}|{c}\oplus {d}$, and hence $F\in {\mathcal {F}}(N)$.

Lastly, suppose that *F* is of Type IV. Without loss of generality, we may assume that $F={a}\oplus {b}\oplus {c}\oplus {d}$. Let $F'$ be the qnet in ${\mathcal {F}}(N)$ whose support is $A=\{a,b,c,d\}$. Hence, there exists no quartet in ${\mathcal {Q}}({\mathcal {F}})$ whose support is *A*. Therefore, $\mathop {med}_T(a,b,c)=\mathop {med}_T(a,b,d)=\mathop {med}_T(a,c,d)=\mathop {med}_T(b,c,d)$. Denoting this median by *u*, it follows that *u* is necessarily contained in $V_2(T)$, and hence $N_T(u)$ contains $m \ge 4$ vertices. Now let $(v_1,v_2,\ldots ,v_m)$ be the unique circular ordering of vertices $N_T(u)$ induced by the circular ordering $A^1,\ldots ,A^m$ of $\mathcal {A}_u$ in Claim 3. Without loss of generality, we may assume that $a\in A^1$. Then, there exists $1<j<k<l\le m$ such that $(b,c,d)\in A^j \times A^k \times A^l$. By the construction of *N* (which locally is the blow-up at *u* with respect to the circular ordering), it follows that $F'$ is isomorphic to *F*, and hence $F\in {\mathcal {F}}(N)$.

This shows that ${\mathcal {F}}\subseteq {\mathcal {F}}(N)$. Since ${\mathcal {F}}$ and ${\mathcal {F}}(N)$ are both minimally dense, we have ${\mathcal {F}}={\mathcal {F}}(N)$. Finally, the uniqueness statement concerning *N* is a direct consequence of the uniqueness of *T* and the unique way in which *N* is constructed from *T*.

## A Characterization of Level-1 Quartet Systems

We now use Theorem 2 to characterize when a quartet system is equal to the set of quartets displayed by a binary level-1 network. This characterization is given as Theorem 3. Let ${\mathcal {Q}}$ be a quartet system on *X*. A quartet *Q* in ${\mathcal {Q}}$ is *distinguished* if *Q* is the only quartet in ${\mathcal {Q}}$ with support equal to the leaf-set of *Q*. Moreover, a network *N* is called 3-cycle free if it does not contain any cycle consisting of three vertices.

### Theorem 3

Let ${\mathcal {Q}}$ be a dense quartet system on *X* with $|X|\ge 4$. Then, ${\mathcal {Q}}={\mathcal {Q}}(N)$ for some binary level-1 network *N* on *X* if and only if the following three conditions hold:

1. For all $Y\in {X \atopwithdelims ()4}$, we have $m_{\mathcal {Q}}(Y)=1$ or $m_{\mathcal {Q}}(Y)=2$.
2. If $\{ab|cd, ad|bc, ax|cd, ac|xd\}\subseteq {\mathcal {Q}}$, then $\{ab|dx,bd|ax\}\subseteq {\mathcal {Q}}$, for $a,b,c,d\in X$ distinct.
3. If *ab*|*cd* is a distinguished quartet in ${\mathcal {Q}}$, then, for each $x\in X-\{a,b,c,d\}$ where $a,b,c,d\in X$ are distinct, either *ax*|*cd* or *ab*|*cx* is a distinguished quartet in ${\mathcal {Q}}$.

Moreover, if ${\mathcal {Q}}$ satisfies (D1)–(D3), then there exists a unique level-1, 3-cycle free network *N* with ${\mathcal {Q}}(N)={\mathcal {Q}}$.

### Proof

It is easily checked that, if ${\mathcal {Q}}={\mathcal {Q}}(N)$ holds for some binary level-1 network *N*, then (D1)–(D3) holds. Conversely, let ${\mathcal {Q}}$ be a dense quartet system satisfying (D1)–(D3). Let ${\mathcal {Q}}_1 \subseteq {\mathcal {Q}}$ be the set consisting of the distinguished quartets contained in ${\mathcal {Q}}$. We first associate a phylogenetic *X*-tree *T* to ${\mathcal {Q}}_1$. If ${\mathcal {Q}}_1=\emptyset$, then we let *T* denote the phylogenetic *X*-tree which contains precisely one vertex that is not a leaf (i.e. a “star tree”). If ${\mathcal {Q}}_1 \ne \emptyset$, then let $Q=ab|cd$ be some quartet contained in ${\mathcal {Q}}_1$, $a,b,c,d\in X$. Suppose that there exists some $x \in X - \{a,b,c,d\}$. Then, by (D3), either $ax|cd \in {\mathcal {Q}}_1$ or $ab|cx \in {\mathcal {Q}}_1$. It follows that $\bigcup _{Q \in {\mathcal {Q}}_1} L(Q) = X$. Moreover, as ${\mathcal {Q}}_1$ is clearly thin and by (D3) ${\mathcal {Q}}_1$ is saturated, it follows by Theorem 1, that there exists a phylogenetic *X*-tree *T* with ${\mathcal {Q}}(T)={\mathcal {Q}}_1$.

Now we construct a qnet system ${\mathcal {F}}$ as follows. Let $\Pi _1$ be the subset of ${X \atopwithdelims ()4}$ consisting of those *Y* with $m_{\mathcal {Q}}(Y)=1$, and $\Pi _2= {X \atopwithdelims ()4} \setminus \Pi _1$. To each $\pi =\{a,b,c,d\} \in \Pi _1$ we associate a qnet $F(\pi )$ as follows. Swapping the labels of the elements in $\pi$ if necessary, we may assume that $Q=ab|cd$ is the (necessarily unique) quartet in ${\mathcal {Q}}_1$ with leaf-set $\pi$. Now let $v_1$ and $v_1'$ be the median of $\{a,b,c\}$ in *Q* and *T*, respectively. Similarly, let $v_2$ and $v_2'$ be the median of $\{a,c,d\}$ in *Q* and *T*, respectively. Then, $F(\pi )$ is the qnet on $\{a,b,c,d\}$ obtained from *Q* by performing a blow-up on each of $v_i$, where $i\in \{1,2\}$, if and only if the degree of $v'_i$ in *T* is at least four.

We also associate a qnet $F(\pi )$ to each $\pi =\{a,b,c,d\} \in \Pi _2$ as follows. Swapping the labels of the elements in $\pi$ if necessary, we may assume that the quartets in ${\mathcal {Q}}$ with leaf-set $\{a,b,c,d\}$ are *ab*|*cd* and *ad*|*bc*. We then define $F(\pi )$ to be the qnet ${a}\oplus {b}\oplus {c}\oplus {d}$.

Now, let $$\mathcal {F}=\{F(\pi ): \pi \in \left( {\begin{array}{c}X\\ 4\end{array}}\right) \}$$. By construction ${\mathcal {F}}$ is minimally dense. Moreover, ${\mathcal {Q}}({\mathcal {F}})={\mathcal {Q}}$, and ${\mathcal {F}}$ is cyclative in view of (D2).

Next, we shall show that ${\mathcal {F}}$ is consistent. Fix a subset $\{a,b,c\} \in {X \atopwithdelims ()3}$ and consider its median *v* in *T*. By construction, it suffices to establish the claim that the degree of *v* is three in *T* if and only if, for each $d\in X-\{a,b,c\}$, the set $\pi =\{a,b,c,d\}$ is not contained in $\Pi _2$.

To see that this claim holds first note that if *v* has degree three, then each of the three components of $T-\{v\}$ contains precisely one element in $\{a,b,c\}$. Without loss of generality, we may assume that element *d* is contained in the connected component containing element *c*. But this implies that *ab*|*cd* is a quartet in ${\mathcal {Q}}(T)$, and hence $\{a,b,c,d\} \in \Pi _1$. On the other hand, if *v* has degree at least four, then there exists an element $x\in X-\{a,b,c\}$ such that *x*, *a*, *b*, *c* belong to four different connected components of $T-\{v\}$. Therefore, ${\mathcal {Q}}(T)$ and $\{ab|cx,ac|bx,ax|bc\}$ are disjoint. This implies that $\pi =\{a,b,c,x\}$ is not contained in $\Pi _1$, and so it is contained in $\Pi _2$. This establishes the claim.

Next, we show that ${\mathcal {F}}$ is saturated. We shall show that (S2) holds; the fact that ${\mathcal {F}}$ satisfies (S1) and (S3) can be established by a similar argument. Let $\{a,b,c,d\} \in {X \atopwithdelims ()4}$ be a set that satisfies the condition in (S2), that is, ${a}\oplus {b}|{c}\ominus {d}$ is contained in ${\mathcal {F}}$. Then, *ab*|*cd* is a quartet in ${\mathcal {Q}}_1={\mathcal {Q}}(T)$. Furthermore, put $u=\mathop {med}_T(a,b,c)$ and $v=\mathop {med}_T(a,c,d)$, then the degree of *u* is at least four and the degree of *v* is three. Now, fix an element $x\in X-\{a,b,c,d\}$. If *x* and *a* are in the same connected component resulting from deleting *v* from *T*, then *ax*|*cd* is a quartet in ${\mathcal {Q}}_1$. Since the median of *a*, *c*, *d* in *T* has degree three, by construction either ${a}\ominus {x}|{c} \ominus {d}$ or ${a}\oplus {x}|{c}\ominus {d}$ (but not both) is contained in ${\mathcal {F}}$. Otherwise, *ab*|*cx* is a quartet in ${\mathcal {Q}}_1$. Since the median *u* of *a*, *b*, *c* in *T* has degree greater than three, by construction we can conclude that either ${a}\oplus {b}|{c}\ominus {x}$ or ${a}\oplus {b}|{c}\oplus {x}$ is contained in ${\mathcal {F}}$ (but not both). This completes the verification of (S2).

It follows that ${\mathcal {F}}$ is minimally dense, cyclative, consistent and saturated. By Theorem 2, there exists a unique binary level-1 network *N* on *X* such that ${\mathcal {F}}(N)={\mathcal {F}}$. By construction, it also follows that ${\mathcal {Q}}(N)={\mathcal {Q}}({\mathcal {F}}(N))={\mathcal {Q}}({\mathcal {F}})={\mathcal {Q}}$. The uniqueness statement in the theorem follows from the uniqueness of *N* and the fact that ${\mathcal {Q}}(N)={\mathcal {Q}}(N')$ for two binary level-1 networks *N* and $N'$ if and only if *N* and $N'$ on *X* differ only by 3-cycles (see e.g. Keijsper and Pendavingh 2014, Lemma 2). $\square$

## Quarnet Inference Rules and Closure

For a quartet system ${\mathcal {Q}}$ on *X*, we write ${\mathcal {Q}}\vdash ab|cd$ precisely if every phylogenetic *X*-tree that displays ${\mathcal {Q}}$ also displays *ab*|*cd*. The statement ${\mathcal {Q}}\vdash ab|cd$ is known as a *quartet inference rule* (Semple and Steel 2003). A well-known example of such a rule is

$$\begin{aligned} \{ab|cd,ac|de\} \vdash ab|ce \end{aligned}$$

which leads to the concept of the *semi-dyadic closure* $\mathrm{cl}_2({\mathcal {Q}})$ of the set ${\mathcal {Q}}$, that is, the minimal set of quartets that contains ${\mathcal {Q}}$ and has the property that if $\{ab|cd,ac|de\} \subseteq \mathrm{cl}_2({\mathcal {Q}})$, then $ab|ce\in \mathrm{cl}_2({\mathcal {Q}})$.

In this section, we define analogous concepts for qnets and show that they have similar properties to those enjoyed by phylogenetic trees. If ${\mathcal {F}}$ is a qnet system, we write ${\mathcal {F}}\vdash F$ for some qnet *F* if every binary level-1 network that displays ${\mathcal {F}}$ also displays *F*. Now, let $*, \diamond ,\circ$ denote symbols in $\{\ominus , \oplus \}$. For example, $a*b | c \diamond d$ is equivalent to $a\ominus b|c \oplus d$ when $*=\ominus$ and $\diamond = \oplus$. We introduce three qnet inference rules on ${\mathcal {F}}$:

1. $$\{a*b | c \diamond d, b\diamond c | d \circ e\} \vdash a *b | c \diamond e$$ for all $$*, \diamond ,\circ \in \{\ominus , \oplus \}$$;
2. $\{a \oplus b | c *d, {a}\oplus {c}\oplus {e}\oplus {b}\} \vdash a\oplus e | c *d$ and $\{a \oplus b | c *d, {a}\oplus {c}\oplus {b}\oplus {e}\} \vdash a\oplus e | c *d$ and $\{a \oplus b | c *d, {a}\oplus {e}\oplus {c}\oplus {b}\} \vdash a\oplus e | c *d\,\,$ for all $*\in \{\ominus , \oplus \}$;
3. $$ \{{a}\oplus {b}\oplus {c}\oplus {d},{e}\oplus {a}\oplus {c}\oplus {d}\} \vdash {a}\oplus {b}\oplus {d}\oplus {e}. $$

**Fig. 5:**
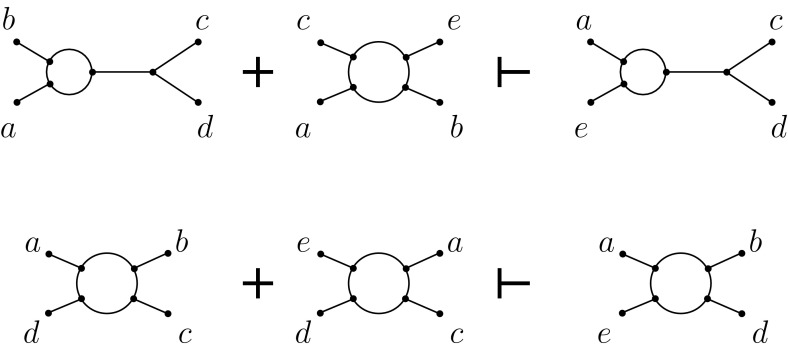
An illustration of the (CL2) and (CL3) inference rules. Top: The first part of the (CL2) inference rule with $*=\ominus$. Bottom: the (CL3) inference rule

We illustrate two of these rules in Fig. 5.

We remark in passing that the qnet system $\{a*b | c \diamond d, b\diamond c | d \circ e\,:\, *, \diamond ,\circ \in \{\ominus , \oplus \} \} \cup \{ a \oplus b | c *d, {a}\oplus {c}\oplus {e}\oplus {b}, {a}\oplus {c}\oplus {b}\oplus {e}, {a}\oplus {e}\oplus {c}\oplus {b}\,:\, *\in \{\ominus , \oplus \} \} \cup \{{a}\oplus {b}\oplus {c}\oplus {d},{e}\oplus {a}\oplus {c}\oplus {d}\}$ implies that inference rules (CL1)–(CL3) are independent from one another.

Using Theorem 2, it is straightforward to show that the above three rules are well defined. That is, given three qnets $F_1$, $F_2$ and *F* such that $\{F_1,F_2\} \vdash F$ holds for one of the above three rules, then every binary level-1 network that displays $\{F_1,F_2\}$ must display *F*.

For a qnet system ${\mathcal {F}}$, we define the set $\mathrm{cl}_2({\mathcal {F}})$ to be the minimal qnet system (under set-inclusion) that contains ${\mathcal {F}}$ such that if $\mathrm{cl}_2({\mathcal {F}}) \vdash F$ holds under (CL1)–(CL3), then $F\in \mathrm{cl}_2({\mathcal {F}})$ holds. We call $\mathrm{cl}_2({\mathcal {F}})$ the *closure* of ${\mathcal {F}}$.

The following key proposition is analogous to that for semi-dyadic closure for quartet systems (cf. Meacham 1983; Huber et al. 2005, Proposition 2.1). It follows from the fact that the closure of a qnet system ${\mathcal {F}}$ can clearly be obtained from ${\mathcal {F}}$ by repeatedly applying the qnet rules (CL1)–(CL3) until the sequence of sets so obtained stabilizes. Note that this process must clearly terminate in polynomial time.

### Proposition 1

Let ${\mathcal {F}}$ be a qnet system and let *N* be a binary, level-1 network. Then, *N* displays ${\mathcal {F}}$ if and only if *N* displays $\mathrm{cl}_2({\mathcal {F}})$.

We now show that $\mathrm{cl}_2({\mathcal {F}})$ behaves in a similar way to the semi-dyadic closure of a quartet system (cf. Semple and Steel 2003, Exercise 19, p. 143).

### Theorem 4

Suppose that ${\mathcal {F}}$ is a minimally dense, consistent set of qnets on *X* with $|X|\ge 5$. Then, the following statements are equivalent:

(i) ${\mathcal {F}}={\mathcal {F}}(N)$ holds for a (necessarily unique) binary, level-1 network *N* on *X*;
(ii) $$\mathrm{cl}_2({\mathcal {F}})={\mathcal {F}}$$;
(iii) For every 3-element subset ${\mathcal {F}}'$ of ${\mathcal {F}}$, the subset ${\mathcal {F}}'$ is displayed by some binary level-1 network on *X*.

### Proof

The fact that (i) implies (ii) and (i) implies (iii) are straightforward. We complete the proof by showing that (ii) implies (i) and (iii) implies (i).

For the proof of (ii) implies (i), suppose that $\mathrm{cl}_2({\mathcal {F}})={\mathcal {F}}$. Note first that by (CL3) ${\mathcal {F}}$ is cyclative. Moreover, ${\mathcal {F}}$ is minimally dense and consistent by assumption. Hence, by Theorem 2, it suffices to show that ${\mathcal {F}}$ is saturated. To this end, let *w*, *x*, *y*, *z*, *t* be five pairwise distinct elements in *X* such that $F=w*x|y \diamond z$ is contained in ${\mathcal {F}}$ with $*,\diamond \in \{\oplus ,\ominus \}$ and $(*,\diamond )\not =(\ominus ,\oplus )$. We need to show that ${\mathcal {F}}$ satisfies (S1)–(S3).

For $p \in \{w,x,y,z\}$, let $F_p$ be the qnet on $\{w,x,y,z,t\}-\{p\}$ that is contained in ${\mathcal {F}}$ (which must exist as ${\mathcal {F}}$ is minimally dense). First assume that there exists some element *p* in $\{w,x,y,z\}$ such that the qnet $F_p$ is of Type IV. Without loss of generality, assume $p=w$ (the other cases can be established in a similar manner). Since $F_w$ is of Type IV, by the consistency of ${\mathcal {F}}$ we have $F=y\oplus z|w*x$. Now, applying (CL2) with $a=y$, $b=z$, $c=w$, $d=x$, $e=t$ implies that $y\oplus t|w *x \in \mathrm{cl}_2({\mathcal {F}})={\mathcal {F}}$, by (ii). Therefore, ${\mathcal {F}}$ satisfies (S2) and (S3) (corresponding, respectively, to taking $*=\ominus$ and $*=\oplus$). It follows that in the remainder of the proof we can assume that none of the qnets in $\{F_w,F_x,F_y,F_z\}$ is of Type IV.

For convenience, in the following, we will use the convention that when we apply (CL1), we will write a 5-tuple and assume that the *i*th element in the 5-tuple will correspond to the *i*th element in the tuple (*a*, *b*, *c*, *d*, *e*) of elements used in (CL1) for $1\le i \le 5$.

To show that ${\mathcal {F}}$ satisfies (S1), suppose that $F={w}\ominus {x}|{y} \ominus {z}$. Note first that if $F_x=w\ominus y|z *t$, then applying (CL1) to (*x*, *w*, *y*, *z*, *t*) implies $x\ominus w |y \ominus t \in \mathrm{cl}_2({\mathcal {F}})={\mathcal {F}}$, and hence (S1) holds. Similarly, if $F_z=w\ominus y|x *t$, then applying (CL1) to (*z*, *y*, *w*, *x*, *t*) implies $z\ominus y |w \ominus t \in {\mathcal {F}}$, and hence (S1) holds. Therefore, if (S1) does not hold, then, by consistency, we may assume $F_x=w\ominus z|y *t$ and $F_z=x\ominus y|w *t$ with $*\in \{\ominus , \oplus \}$. Considering $F_x$ and $F_z$, and applying (CL1) to (*x*, *y*, *t*, *w*, *z*) implies $x\ominus y | t *z \in {\mathcal {F}}$. On the other hand, considering *F* and $F_z$ and applying (CL1) to (*z*, *y*, *x*, *w*, *t*) implies that $z\ominus y | x \ominus t \in {\mathcal {F}}$, a contradiction to the fact that ${\mathcal {F}}$ is minimally dense. Thus, ${\mathcal {F}}$ satisfies (S1).

Using an argument similar to the one that we used to show that ${\mathcal {F}}$ satisfies (S1), it is straightforward to deduce that ${\mathcal {F}}$ satisfies (S2) and (S3).

We next prove that (iii) implies (i). Since ${\mathcal {F}}$ is minimally dense and consistent by assumption, it follows by Theorem 2 that it suffices to show that ${\mathcal {F}}$ is cyclative and saturated.

First, we show that ${\mathcal {F}}$ is cyclative. If not, then there exist five elements $Y=\{w,x,y,z,t\}$ such that $F_1={w}\oplus {x}\oplus {y}\oplus {z}$ and $F_2={t}\oplus {w}\oplus {y}\oplus {z}$ are contained in ${\mathcal {F}}$ but $F={w}\oplus {x}\oplus {z}\oplus {t}$ is not contained in ${\mathcal {F}}$. Let $F'$ be the (necessarily unique) qnet in ${\mathcal {F}}$ whose leaf-set is $\{w,x,z,t\}$. Then, $F'\not = F$. Consider the set ${\mathcal {F}}'=\{F',F_1,F_2\}$. The assumption (iii) implies that ${\mathcal {F}}'$ is displayed by a binary level-1 network *N* on *X*. Consider $N'=N|_Y$. Then, ${\mathcal {F}}'\subseteq {\mathcal {F}}(N')$. By Theorem 2, ${\mathcal {F}}(N')$ is minimally dense and cyclative. Since $\{F_1,F_2\}\subseteq {\mathcal {F}}(N')$, it follows that $F\in {\mathcal {F}}(N')$, a contradiction in view of $F' \in {\mathcal {F}}(N')$.

Second we show that ${\mathcal {F}}$ is saturated. Here, we only show that ${\mathcal {F}}$ satisfies (S2) as showing that ${\mathcal {F}}$ satisfies (S1) and (S3) can be done in a similar manner. If ${\mathcal {F}}$ does not satisfy (S2), then there exists a 5-element set $Y=\{w,x,y,z,t\}$ such that $F={w}\oplus {x}|{y}\ominus {z}$ is contained in ${\mathcal {F}}$ while, for the qnet system

$$\begin{aligned} {\mathcal {F}}^* = \{{w}\oplus {x}|{y}\ominus {t}, {w}\oplus {x}|{y}\oplus {t}, {w}\ominus {t}|{y} \ominus {z}, {w}\oplus {t}|{y}\ominus {z} \}, \end{aligned}$$

we have ${\mathcal {F}}^*\cap {\mathcal {F}}=\emptyset$. Let $F_1$ and $F_2$ be the qnets in ${\mathcal {F}}$ with leaf sets $A=\{w,x,y,t\}$ and $B=\{w,t,y,z\}$, respectively which must exist as ${\mathcal {F}}$ is minimally dense by assumption. Then, neither $F_1$ nor $F_2$ is contained in ${\mathcal {F}}^*$.

Lastly, consider the subset ${\mathcal {F}}'=\{F,F_1,F_2\}$ of ${\mathcal {F}}$. Then, as assumption (iii) holds it follows that ${\mathcal {F}}'$ is displayed by a binary level-1 network *N* on *X*. Consider $N'=N|_Y$. Then, ${\mathcal {F}}'\subseteq {\mathcal {F}}(N')$. By Theorem 2, ${\mathcal {F}}(N')$ is minimally dense and saturated. Using the fact that ${\mathcal {F}}(N')$ is saturated, it follows that ${\mathcal {F}}^*\cap {\mathcal {F}}(N') \not = \emptyset$ as $F \in {\mathcal {F}}(N')$. Therefore, ${\mathcal {F}}(N')$ contains either two distinct qnets on *A* or two distinct qnets on *B*, a contradiction to the fact that ${\mathcal {F}}(N')$ is minimally dense. Thus, (iii) implies (i), thereby completing the proof of the theorem. $\square$

Note that it follows from Theorem 4 that we can decide whether or not a given minimally dense set of qnets ${\mathcal {F}}$ is displayed by a level-1 binary phylogenetic network on $n\ge 2$ leaves in $O(n^5)$ time. This follows since we can compute $\mathrm{cl}_2({\mathcal {F}})$ in $O(n^5)$ time. It would be interesting to see if this time bound can be improved upon.

## Discussion

We have shown that by considering quarnets we can define natural inference rules, as well as the concept of quarnet closure. With quartets, there are various types of inference rules, which imply alternative definitions of closure for quartet systems (see e.g. Bryant and Steel 1995; Semple and Steel 2003). It would thus be of interest to explore whether there are other types of inference rules for quarnets and, if so, what their properties are. In this paper, we have focused on understanding the closure for a minimally dense set of quarnets. For real data, there can be cases where it may be necessary to consider non-minimally dense sets (e.g. in case there is missing data). Hence, it could be useful to develop results for such situations. However, it should be noted that understanding the closure of a non-minimally dense set quartets is already quite challenging (for example, as opposed to the minimally dense case, deciding whether or not an arbitrary set of quartets can be displayed by a phylogenetic tree is NP-complete) (Steel 1992).

In many applications, biologists prefer to use weighted phylogenetic trees and networks to model their data, where non-negative numbers are assigned to edges of the tree or network to, for example, represent evolutionary distance. The problem of considering when a dense set of weighted quartets can be represented by a weighted phylogenetic tree has been considered in Dress and Erdös (2003), Grünewald et al. (2008). Given the results in this paper, it could therefore be of interest to consider how weighted level-1 networks may be inferred from dense sets of weighted quarnets. In applications, it can also be useful to consider rooted networks, which are essentially leaf-labelled, directed acyclic graphs. Edges in such networks have a direction which represents the fact that species evolve through time from a common ancestor (represented in graph theoretical terms by a root vertex). For such networks, the concept of level-1 networks can be defined in a similar way to the unrooted case, and algorithms are known for deciding when minimally dense collections of 3-leaved, rooted level-1 phylogenetic networks (which are known as *trinets*) can be displayed by a single phylogenetic network (Huber and Moulton 2013; Huber et al. 2017). It would thus be of interest to consider inference rules for trinets. Moreover, for both the rooted and unrooted case, it could be worth exploring whether there are inference rules for more complicated networks (e.g. networks with level higher than one, as defined in e.g. Gambette et al. 2012). Although results in Iersel and Moulton (2017) indicate that such inference rules might exist, if they do, then we expect that these will probably be quite complicated.

## Footnotes

1 Note that this concept was first introduced for *rooted* networks—see Jansson and Sung (2004) for more details

2 Note that this notion of a qnet is not related to the notion of a qnet introduced in Grünewald et al. (2007)
